# Leptin and Obesity: Role and Clinical Implication

**DOI:** 10.3389/fendo.2021.585887

**Published:** 2021-05-18

**Authors:** Milan Obradovic, Emina Sudar-Milovanovic, Sanja Soskic, Magbubah Essack, Swati Arya, Alan J. Stewart, Takashi Gojobori, Esma R. Isenovic

**Affiliations:** ^1^ Department of Radiobiology and Molecular Genetics, “VINČA” Institute of Nuclear Sciences - National Institute of the Republic of Serbia, University of Belgrade, Belgrade, Serbia; ^2^ Computer, Electrical and Mathematical Sciences and Engineering Division (CEMSE), Computational Bioscience Research Center, Computer (CBRC), King Abdullah University of Science and Technology (KAUST), Thuwal, Saudi Arabia; ^3^ School of Medicine, University of St Andrews, St Andrews, United Kingdom; ^4^ Biological and Environmental Sciences and Engineering Division (BESE), King Abdullah University of Science and Technology (KAUST), Thuwal, Saudi Arabia

**Keywords:** leptin-based therapies, obesity, leptin resistance, leptin receptor, leptin

## Abstract

The peptide hormone leptin regulates food intake, body mass, and reproductive function and plays a role in fetal growth, proinflammatory immune responses, angiogenesis and lipolysis. Leptin is a product of the obese (*ob*) gene and, following synthesis and secretion from fat cells in white adipose tissue, binds to and activates its cognate receptor, the leptin receptor (LEP-R). LEP-R distribution facilitates leptin’s pleiotropic effects, playing a crucial role in regulating body mass *via* a negative feedback mechanism between adipose tissue and the hypothalamus. Leptin resistance is characterized by reduced satiety, over-consumption of nutrients, and increased total body mass. Often this leads to obesity, which reduces the effectiveness of using exogenous leptin as a therapeutic agent. Thus, combining leptin therapies with leptin sensitizers may help overcome such resistance and, consequently, obesity. This review examines recent data obtained from human and animal studies related to leptin, its role in obesity, and its usefulness in obesity treatment.

## Introduction

Obesity-associated co-morbidities such as hypertension, dyslipidemia, type 2 diabetes mellitus, fatty liver disease, heart disease, and some types of cancer cause about 3.4 million adults (over age 18) deaths in 2016, according to the World Health Organization (1). They further reported that an alarming 1.9 billion adults are overweight, and over 650 million overweight adults are obese. Hyperleptinemia and resistance to a reduction of body mass are two common characteristics of obesity (2). In this regard, studies report a strong positive association between serum leptin levels and the percentage of body fat (3, 4). Thus, pharmaceutical companies are pursuing the idea of using leptin-based drugs as a therapeutic strategy for weight loss (5, 6).

In 1994 Zhang et al. identified leptin as the product of the obese (*ob*) gene after characterizing genetically obese (*ob/ob*) mice (7). This factor was coined leptin the following year, derived from the Greek word *leptos*, meaning thin (8). Leptin regulates food intake, body mass, reproductive functioning and plays a vital role in fetal growth, proinflammatory immune responses, angiogenesis, and lipolysis (2, 9, 10). Studies demonstrated that the concentration of circulating leptin decreases during fasting (11) or energy restriction (12) but increases during refeeding (13), overfeeding (14), as well as during surgical stress (15). These effects provide an overview of how various pathways regulate the leptin signaling system to maintain body mass. For example, when the fat cells increase, leptin levels increase proportionally, then bind to leptin receptors (LEP-R) in the brain that send signals to inhibit food intake and increase energy expenditure (16, 17). However, when a positive energy balance (i.e., caloric intake exceeds energy expenditure) is sustained for critical periods, weight is gained (3, 16, 17). Here we review the literature to collate and provide a comprehensive summary of the relationship between leptin signaling and obesity.

## Leptin and Its Cognate Receptor

The leptin molecule is 16 kDa in size and comprises 167 amino acids (including a 21 amino acid secretory signal sequence), and it exhibits the tertiary structure of a globular protein (18, 19). Leptin acts *via* its transmembrane receptors, the LEP-R, that exhibit structural similarity to the class I family of cytokine receptors, which include receptors for interleukins (IL), leukemia inhibitory factor (LIF), colony-stimulating factor 3 (CSF-3), growth hormone (GH), prolactin and erythropoietin (20–23). These family members have characteristic extracellular motifs, including four cysteine residues, a Trp-Ser-Xaa-Trp-Ser motif, and fibronectin type III (FN III) domains (24). LEP-R exists in several alternatively spliced variants labeled as LEP-Ra, LEP-Rb, LEP-Rc, LEP-Rd, LEP-Re, and LEP-Rf and the extracellular and transmembrane domains comprise over 800-amino acids and 34-amino acid, respectively, while a variable intracellular domain characteristic for each of the LEP-R isoforms (21–23, 25). The isoforms are classified into three classes: short, long, and secretive (23).

### The Role of Leptin in the Regulation of Energy Balance

Brain lesion and stimulation research led to the discovery of the “satiety center” in the ventromedial hypothalamic nucleus (VMH) and the “hunger center” in the lateral hypothalamic nuclei (LH). This defines the dual-center model for feeding, proposing that energy input is provided through eating (26). Thus, energy balance is maintained when energy from food intake is equal to energy expenditure. About one year after the discovery of the leptin gene, it is shown that leptin regulates appetite and metabolism by inhibiting the synthesis and release of neuropeptide Y (NPY) in the arcuate nucleus (ARC) (27). Subsequently, it was discovered the LEP-R isoform b (LEP-Rb) in the VMH, ARC, LH, and the dorsomedial hypothalamic nucleus (DMH), which plays a crucial role in the regulation of energy balance and body mass (28). Earlier studies revealed that lesions of the ARC, VMH, or DMH could lead to hyperphagia and obesity in rats (29, 30), and lesions of the LH can lead to anaphylaxis (31). Later studies have demonstrated that leptin can inhibit neural pathways activated by appetite stimulants (orexigenic) to reduce energy intake and activate pathways targeted by anorexigenic to suppress appetite (32, 33). Examples of orexigenic neuropeptides include NPY and the agouti-related protein (AgRP). The product of proopiomelanocortin (POMC), alpha-melanocyte-stimulating hormone (α-MSH), is an anorexigenic (34). Neurons that express AgRP, POMC, and melanocortin include those in the central melanocortin system involved in energy balance regulation (34, 35).

**Figure 1:**
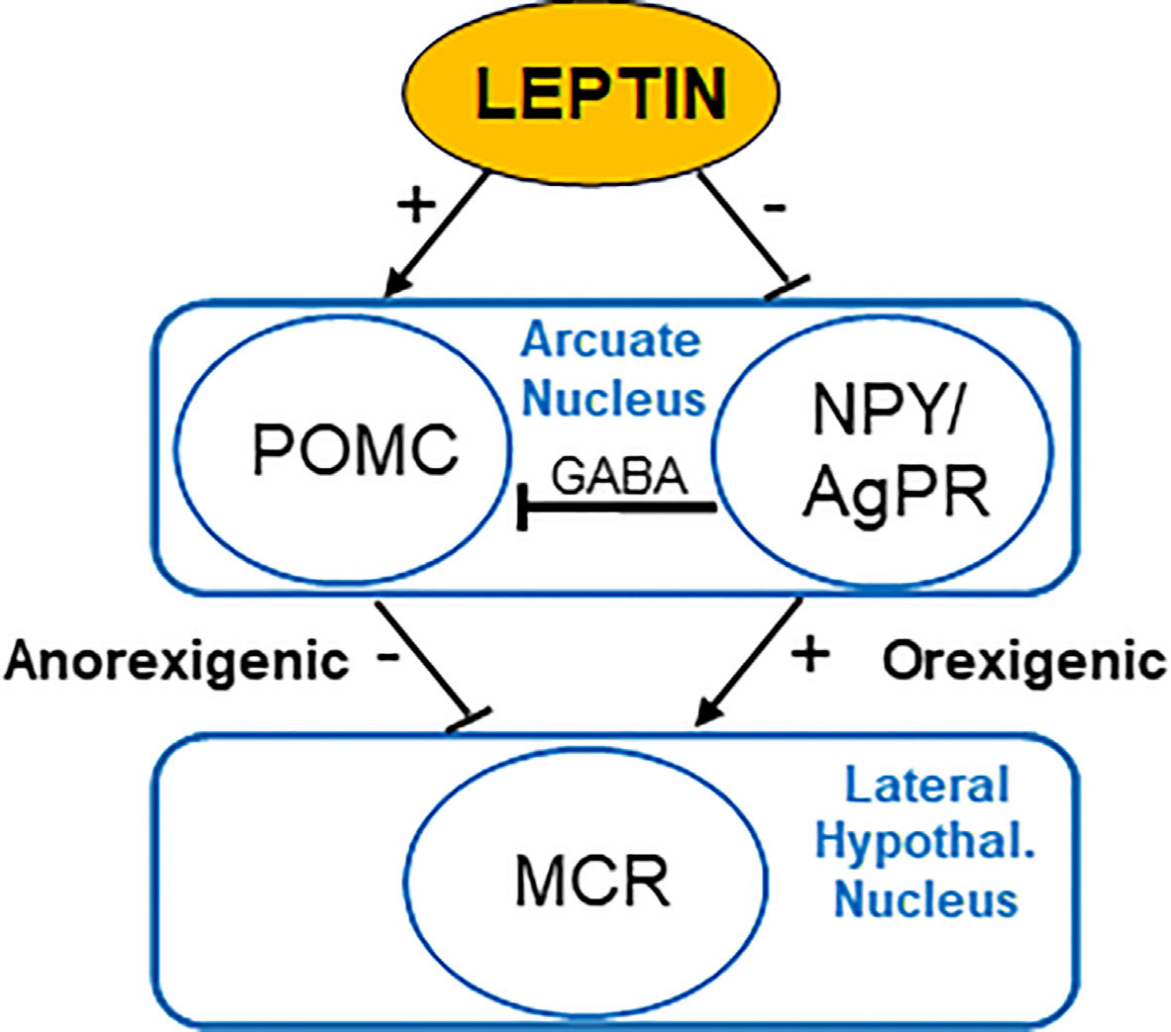
Regulation of appetite by leptin acting on the nucleus arcuatus of the hypothalamus. POMC, proopiomelanocortin; NPY, neuropeptide Y; AgRP, agouti-related protein; MCR, melanocortin receptors; GABA, γ-aminobutyric acid.

The interaction between the signaling of leptin and the dominant feeding regulation constitutes a simple model: leptin affects the transcription of POMC, whose α-MSH product is released into the synapse to activate neurons *via* binding to the melanocortin receptor (MCR) and leads to appetite-suppression (36, 37). Also, leptin inhibits NPY/AgRP synthesis in neurons, which, in turn, reduces the agonistic effect of AgRP on MCR (**Figure 1**) (36, 37).

The significance of the melanocortin system is not only due to the direct action of leptin in the hypothalamus but also the fact that the loss of melanocortin 4 receptor (MC4R) function, a key MCR expressed in the hypothalamus, is the most common genetic cause of obesity in humans and occurs in 3-5% people with extreme obesity (38, 39). In brief, leptin regulates energy balance by modulating the activity of NPY/AgRP and POMC neurons in the ARC nucleus (34). Another mechanism of energy balance regulation was discovered by identifying rapid regeneration of the ARC nucleus’ neural circuits using leptin (40). Among *ob/ob* mice and wild-type mice are different synapses extended on NPY/AgRP and POMC neurons (40). Furthermore, leptin treatment normalized the synaptic density on NPY/AgRP and POMC neurons 6 hours after treatment, a few hours before it affected food intake (40). These findings indicate that leptin acts on the hypothalamus by regulating neuronal plasticity (34, 41).

### Regulation of Leptin Secretion

Leptin is primarily produced in white adipose tissue. Still, smaller quantities have been detected in other body tissues, including the brown adipose tissue (BAT), placenta, fetal tissue, stomach, muscles, bone marrow, teeth, and brain (42, 43). Leptin circulates in the blood in both free and protein-bound forms, where the free form of leptin is the biologically active form (43). The equilibrium between free and bound leptin regulates leptin bioavailability (39). Leptin can enter the central nervous system (CNS) (in the area of the choroid plexus) by receptor-mediated transport (44). The LEP-R isoform plays a particularly significant role in transporting leptin through the blood-brain barrier (BBB) (44). A complex array of endocrine, neuroendocrine, and paracrine signals governs leptin synthesis and secretion (45). The secretion of leptin is proportional to body mass and nutritional status. The serum leptin levels decrease during starvation, associated with an adaptive physiological response to the state of starvation (45). Furthermore, leptin secretion is higher in subcutaneous than in visceral adipose tissue (46, 47).

Food intake, total body fat, as well as several hormones regulate leptin secretion (45). Insulin and, to a lesser extent, other pancreatic peptide hormones, including amylin, glucagon, and pancreatic polypeptides, reduce food intake and affect leptin secretion (48). Insulin is the primary regulator of leptin production (49). Prolonged hyperinsulinemia leads to an increase in leptin’s plasma concentration, while short-term hyperinsulinemia does not cause such a change (49). Moreover, insulin infusion increases plasma leptin concentration in humans (50), and rodents with type 1 diabetes exhibit significantly reduced leptin levels (51). Based on such *in vitro* studies, it is assumed that insulin stimulates leptin production *via* glucose metabolism (51–53). The blockade of glucose transports or glycolysis in the presence of high insulin levels inhibits the expression and secretion of leptin in adipocytes (51, 53). Changes in glucose metabolism due to the application of a high-fat diet for 24 hours explain the reduced level of leptin in human circulation and thus contribute to a high-fat diet in promoting weight gain and obesity (54). The reduced level of leptin in the circulation observed during high energy consumption is associated with humans’ hunger (45). Therefore, leptin flows from the adipocyte into the bloodstream, passes through the BBB, and arrives in areas of the brain involved in regulating the hypothalamus’s energy balance (55). Unlike insulin, catecholamines bind to β2- and β3-adrenergic receptors to inhibit leptin synthesis (52), indicating a link between neuroendocrine and sympathetic control of adipose tissue endocrine function, i.e., the existence of negative feedback between the brain and adipose tissue (56). Corticosteroids and tumor necrosis factor α (TNF-α) stimulate leptin synthesis, while thyroid hormones are likely to decrease it (49).

### Molecular Mechanisms of Leptin Action

**Figure 2:**
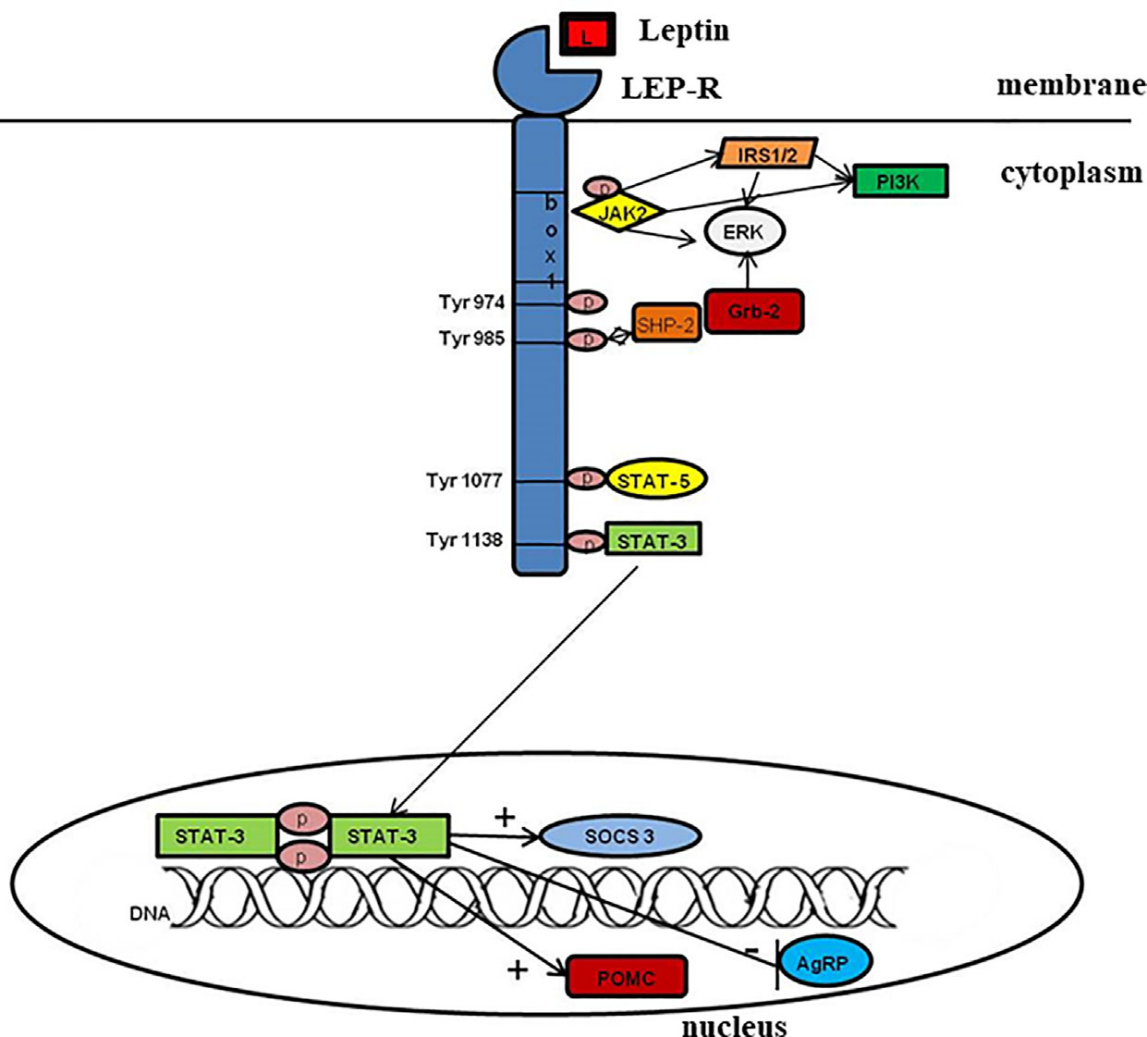
Leptin signaling. L- leptin; LEP-R- leptin receptor; IRS 1/2, insulin receptor substrate 1/2; JAK 2, Janus kinase 2; PI3K, phosphoinositide 3-kinase; SH2, Src-like homology 2; SHP-2, SH2 domain-containing protein tyrosine phosphatase; SOCS3, suppressor of cytokine signaling 3; STAT, signal transducer and activator of transcription.

The distribution of the LEP-R facilitates the pleiotropic effects of leptin (23). The binding of leptin to its receptor initiates numerous signal transduction pathways and, as a result, regulates a range of cellular functions in the body (19, 23). LEP-R, as a member of the type I cytokine receptor family, signals *via* the Janus kinase family (**Figure 2**) of tyrosine kinases (57). The intracellular domain of all LEP-R isoforms contains in the juxtamembrane region a “box” 1 -JAK-binding domain, while LEP-Rb also includes a “box” 2 motif and a signal transducer and activator of transcription (STAT)-binding sites (23, 58–60). Usually, functional receptors for cytokines contain the box 1 motif required for the interaction and activation of JAK (61). Box 2 also plays a role in the interactions and selectivity of JAK isoforms. However, for leptin signaling, only box 1 and an Ala-Ala motif in the immediate environment are essential for JAK activation (62, 63). Although initially only LEP-Rb was observed as an isoform involved in signaling, it has also been demonstrated for the short isoforms (64–66). Mainly JAK2 members of the JAK family proteins are associated with membrane-proximal sequences of the intracellular receptor domain, which is phosphorylated after binding the ligand.

LEP-R and other cytokine receptors do not have kinase activity but couples with tyrosine kinases. After LEP-R binds leptin, LEP-R undergoes a conformational change, critical for leptin signaling and activation of the associated JAK2. JAK2 autophosphorylates and simultaneously phosphorylates tyrosine residues on the functional LEP-R’s intracellular domain, allowing binding of STAT proteins and their subsequent translocation to the nucleus where they act as transcription factors (23). Also, cytokine signaling 3 (SOCS3) and protein tyrosine phosphatase 1B (PTP1B) can act as suppressors of the JAK-STAT pathway (23, 67, 68). PTP1B is a known negative modulator of leptin signal transduction *via* the de-phosphorylation of JAK2. Excessive expression of PTPB1 reduces phosphorylation of JAK2 and inhibits the transcription of SOCS3 and c-fos, which are induced by leptin (23). Furthermore, isoforms of LEP-R with a long intracellular domain may also activate other signaling pathways. The binding of leptin to LEP-R also activates phosphoinositol-3 kinase (PI3K) (69) and mitogen-activated protein kinases/extracellular signal-regulated kinase (MAPK/ERK) (70) signaling cascades. The activation of each of these pathways contributes to leptin’s anorexigenic effects (suppressing appetite, stimulating weight loss, and increasing thermogenesis) (69–71).

It is important to note that distinct signal transduction pathways are responsible for mediating the leptin’s metabolic effects compared with its cardiovascular effects. For example, the JAK2/STAT3 pathway is primarily responsible for regulating gene expression changes, while the PI3K pathway often signals more rapidly through phosphorylation of cytoplasmic proteins. The PI3K pathway plays an important role in leptin’s acute effects, such as regulating food intake and arterial hypertension (72). However, the Jak/STAT3, MAPK, and PI3K pathways appear to collectively regulate energy balance (72).

The effects of leptin are similar to other acute phase reactants; it increases the secretion of multiple inflammatory cytokines such as IL-6, IL-12, and TNF-α (73). In turn, exposure to inflammatory stimuli such as TNF-α and IL-1 increases leptin expression in the adipose tissue and circulating leptin, which creates a feedback loop that promotes inflammation (74, 75). This feedback loop emphasizes how leptin promotes low-grade inflammation since the proinflammatory mediators increase leptin expression and other acute phase reactants that promote chronic inflammation.

The effects of leptin are manifold; it stimulates the expression of IL-1Rα, a cluster of differentiation (CD) 25, CD39, CD69, and CD71 (76), and the production of proinflammatory cytokines TNF-α and IL-6 (77) in macrophages. The number of macrophages present in white adipose tissue correlates directly with obesity, i.e., obese individuals have more macrophages in adipose tissue (78, 79). The adipocyte-produced cytokines, CC-chemokine ligand 2 (CCL2), contribute to this macrophage infiltration process. The macrophages and adipocytes in adipose tissue are major TNFα and IL-6 sources in obese individuals, respectively. Thus, together these adipose tissue cells are also involved in a feedback loop that perpetuates macrophage recruitment and production of proinflammatory cytokines. These feedback loops explain why obesity is associated with chronic pro-inflammatory signaling pathways, abnormal cytokine production, and increased acute-phase reactants (80) and why obesity increases an individual’s risk of developing inflammatory-based diseases and immune-mediated disorders (80–82).

## Leptin and Obesity

### Leptin Expression in Obesity

Severe early obesity develops from rare genetic mutations that affect leptin signaling (2, 83). Such mutations often lead to congenital leptin deficiency or high but ineffective leptin and leptin resistance (84). Hyperleptinemia and resistance to reducing body mass are two characteristics of typical obesity (2, 3, 85). Leptin is overexpressed at the gene level in the adipose tissue of individuals with obesity (86). Furthermore, strong positive associations exist between plasma leptin levels and body fat percentage (87, 88). Other studies point towards leptin resistance. For example, plasma leptin levels and *ob* mRNA content decrease in individuals with obesity at the initial time of weight loss but increases as they continue to lose weight (88). Also, despite the expectation, leptin therapy’s termination does not result in weight gain and hyperleptinemia (89). There is also evidence that hyperleptinemia does not mimic the CNS consequences of chronic weight gain in diet-induced obese (DIO) mice (2, 89).

Different areas within the brain may be involved in the temporal and spatial dysregulation of neurological functioning associated with leptin under nutrient excess conditions (90). In this regard, Matheny et al. demonstrated that consuming a diet rich in high-fat induced leptin resistance in the ARC and ventral tegmental area (VTA), while medial basal hypothalamic regions stayed sensitive to leptin (91). Subsequently, the selective downregulation of Ob-Rb using lentivirus in ARC promoted diet-induced obesity in rats (92), demonstrating the ARC region has a role when leptin resistance develops in obesity. Interestingly, DIO is induced by the differential expression of leptin in brain regions, which may result from the various experimental methods used to regulate leptin expression. Moreover, these studies show the anorectic effects of leptin are not brain-specific. The ARC and VTA appear to be the main areas for the responsiveness of leptin. When the response to leptin is decreased in one region of the brain, it may be overcompensated by another, which suggests coordinated functioning. A high-fat diet may cause SOCS3 expression and activation of STAT3 resistance by leptin in POMC (93), ARC (94, 95), and AgRP neurons in rodents. Also, in AgRP neurons, the expression of SOCS3 decreases after shifting from high-fat to low-fat diets, indicating that those neurons may be more sensitive to leptin than POMC neurons (90, 96).

Experiments on obese mice confirmed a polymorphism in the *ob* gene (97, 98). This polymorphism alters the leptin protein function such that mice become morbidly obese (97, 98). Similarly, mice with a polymorphism in the gene encoding LEP-R, display altered leptin signaling that leads to obesity (99, 100). A single-nucleotide polymorphism identified in the 5’-untranslated region of the leptin gene (LEP -2548 G/A polymorphism) and its association with obesity is the most studied in humans. Still, the literature data are inconsistent (101–105). Carayol et al. designed and performed the first protein quantitative trait locus (pQTL) analysis in obesity and examined the role of genetic variations in determining protein level variation (106). They identified cis-pQTL and trans-pQTL signals associated with BMI at baseline and after the intervention and concluded that in human adipose tissue, human NTases belonging to the FAM46A family (family-with-sequence-similarity-46) was a negative regulator of leptin signaling (106).

A range of studies has investigated genetic and epigenetic factors that control leptin expression. For example, a distant leptin enhancer 1 (LE1) sequence has been identified 16 kb upstream from the transcription start site (TSS) of the *ob* gene. The LE1 contains a 17-bp non-canonical peroxisome proliferator-activated receptor gamma (PPARγ)/retinoid X receptor alpha (RXRα)-binding site, named leptin regulatory element 1 (LepRE1) that is essential for fat-regulated expression (107). In the same study, a functionally analogous LepRE1 site was also found in a second DNA regulatory element 13 kb downstream from the TSS of the *ob* gene. Non-coding RNAs have been implicated in the regulation of leptin gene expression, with its dysregulation linked to obesity (108) and in the development of hypothalamic leptin insensitivity (109). In addition, leptin has been shown to modulate the expression of miRNAs that target POMC mRNA (110). Epigenetic mechanisms linked to obesity that impact leptin and LEP-R expression are also at play. A study investigating DNA methylation in promoter sequences in bariatric surgery patients found higher *Ob* gene promoter methylation patterns in pre bariatric surgery patients compared to postoperative patients. Whilst DNA methylation of the LEP-R gene promoter was significantly higher in the postoperative group (111).

### Leptin Resistance in Obesity

The term “leptin resistance” was coined shortly after discovering leptin in 1994 (7, 112, 113). The concept of leptin resistance implies the processes that result from a state of obesity impair the effects of leptin, thereby contributing to the formation of obesity and obstructing the potential efficacy of therapy with the use of exogenous leptin (3, 113). Leptin resistance occurs due to the leptin’s inability to reach the target cells, reduced LEP-R expression, or disturbed LEP-R signaling (3, 113). There are likely a number of molecular and genetic mechanisms that can lead to leptin resistance. Although relatively rare, loss of function mutations has been identified in genes encoding leptin and its receptors (28, 114, 115). More common mechanisms likely include defects in the pathways that regulate leptin synthesis. Leptin concentrations are directly dependent upon *Ob* gene transcription, which correlates with adipocyte size and lipid content. A complete understanding of how these factors are mechanistically linked or how such pathways are altered to trigger leptin resistance remains unclear. However, additional external stimuli, including eating behaviors and the circadian rhythm, modulate leptin expression and may play a role (116). It has been demonstrated that the decreased transport of leptin across BBB can lead to leptin resistance. Microcapillary vessels at the BBB express short truncated LEP-R forms that bind leptin and transport it to the nervous system (21, 117). It has been shown that even if plasma leptin levels rise above the range of 25–30 ng/mL, the concentration of leptin in cerebrospinal fluid does not increase further (118). Furthermore, it appears that excessive plasma leptin levels can result in decreased BBB permeability (119, 120).

A more nuanced or selective form of leptin resistance (SLR) has also been described, where the effects of leptin on appetite (and body mass) are absent. Still, the results of leptin on the sympathetic nervous system are preserved (3, 113). Interestingly, SLR characterizes preservation of sympathetic nerve activity (SNA) in the kidney and normal blood pressure (BP) responses to leptin action in obesity, despite alterations in responses to leptin in appetite, thermogenesis, and body mass (121). Two potential overlapping pathogenic mechanisms for SLR development have been proposed. Firstly, defects in differential leptin molecular signaling pathways that mediate selective as opposed to universal leptin action and secondly, defects in processes that regulate brain site-specific leptin actions (121).

Moreover, the latest studies unexpectedly propose that the brain renin-angiotensin system (RAS) mediates the leptin effects on renal and BAT thermogenic SNA with the absence of the effects of leptin on food intake (121). These findings imply that elevation or reduction of brain RAS activity may regulate leptin actions on BP and energy expenditure with no impact on the leptin-induced reduction in food intake (121). BAT thermogenesis is stimulated by leptin *via* central LEP-R, acting primarily through the sympathetic nervous system (122–124). A few hypothalamic areas (DMH, preoptic area (POA), paraventricular nucleus (PVN), VMH, ARC), but also some extra-hypothalamic regions as the nucleus tractus solitarius (NTS) participate in leptin-induced thermogenesis (125). The sympathetic regulation of BAT implicates neurons of the NTS that obtain vagal information and project nearby in the hypothalamic areas and the brainstem (126). Since NTS neurons have LEP-R, a specific administration of leptin to NTS leads to a decline of body mass accompanied with a decrease in food intake (124, 127).

Besides leptin actions/resistance on neurons in the hypothalamus, an SLR that extends to some extra-hypothalamic brain areas has been described. SLR in ARC of DIO mice has been shown, whereas other hypothalamic and extrahypothalamic nuclei remain leptin responsive (33, 95). Although DIO induced site-specific leptin resistance, constant overexpression of leptin in CNS induced leptin resistance in every brain region investigated. This suggests that SLR is distinctive to DIO and is not a nonspecific central neural response induced by high leptin exposure (91, 121).

Furthermore, in contrast to insulin, which induces improvement in SNA by acting in ARC as the only specific site, leptin takes action in few hypothalamic sites, all of which seem to interact in PVN (128). The main effects of insulin and leptin in states of obesity include sexually dimorphic alterations. The latest observations regarding the link between sexual dimorphism and sympathetic in the obese human population reveal that several variations exist in lean females that restrict the effects of leptin and insulin to increase SNA and/or BP (128). The first is that only during proestrus leptin increases SNA by the synergistic effects of raised concentrations of estrogen. The second one is that leptin and insulin do not induce the rise in SNA, leading to vasoconstriction and BP elevation in females while induced in males (128).

Furthermore, in obese males, sympathoexcitatory response to insulin is increased, unlike in obese females, where it is eliminated. Regarding leptin and its sympathoexcitatory response, it is also preserved or increased in obese males. In contrast, in obese females, the reproductive cycle is disturbed, and the sympathoexcitatory response to leptin is limited. This is probably due to the sexually dimorphic changes in NPY and POMC entrants to PVN. In obese males, stimulant PVN and NPY sympathoinhibitory response is abolished, and POMC entrant to PVN is elevated, probably due to increased cellular signaling of ARC and POMC induced by insulin.

Conversely, in obese females, stimulant NPY sympathoinhibitory response is preserved and not inhibited by insulin, and POMC insulin sensitivity may also be reduced. Until now, the mechanisms for obesity-induced sexually dimorphic alterations are not fully elucidated. There is a hypothesis that a considerable suppression of NPY *via* hypertensive, as opposed to a non-hypertensive branch of RAS, and a considerable POMC excitation, in obese males concerning obese females might be important. Nonetheless, the precise mechanisms in the base of insulin and leptin actions on ARC, NPY, or POMC and silenced in obese females have not yet been fully discovered (128).

### Clinical Trials Examining the Effectiveness of Leptin-Based Interventions in Obesity

Combining therapies of leptin and leptin sensitizers can overcome leptin resistance (16, 129). **Table 1** summarizes some essential clinical trials investigating the use of such agents. The first clinical study observing common polygenic or simple obesity with recombinant methionyl human leptin (r-metHuLeptin), also known as metreleptin, was carried out by Heymsfield and colleagues in 1999 (130). As the leptin dose increases, the group with obesity exhibits mean weight changes ranging from 0.7 kg to 7.1 kg over 24 weeks (130). The administration of pegylated human recombinant leptin (PEG-OB) was studied in men with obesity (131, 132) using weekly doses combined with a moderate diet. These pilot 12-week clinical studies demonstrated no difference in weight between the PEG-OB group and a placebo group (131, 132).

**Table 1:** Summary of some clinical trials involving the use of leptin-based therapies to treat obesity.

Study design	Subjects	Leptin level before therapy (ng/ml)	Treatment/Drug	Effects in the group with obesity	Ref.
randomized, double-blind, placebo-controlled, multicenter, escalating dose cohort trial in both lean and obese adults, over 24 weeks	54 lean, 73 obese; 67 men, 60 women	37 have 15.9; 16 have 16.8; 16 have 16.4; 31 have 12.3; 26 have 15.1	r-metHuLeptin, daily morning subcutaneous injection	• weight loss with a mean of -7.1 kg after 24 weeks• weight loss caused by r-metHuLeptin may be due almost entirely to fat loss	(130)
a randomized, double-blind, placebo-controlled trial in obese men, over 12 weeks	30 obese men; 15 from 30-placebo; 15 from 30-PEG-OB	Placebo group: 20.4 ± 4.9; PEG-OB group: 20.4 ± 4.9	PEG-OB 20 mg/once a weekly subcutaneous injection in combination with a moderate diet	• weight loss, body fat reduction, total energy expenditure, or sleeping metabolic rate differences were non-significant when comparing the PEG-OB group with the placebo group	(131, 132)
a randomized, double-blind, placebo-controlled study in overweight men, over 46 days and a follow-up for 2 weeks	24 overweight men; 12 from 24-placebo; 12 from 24-PEG-OB	Placebo group: 7.3 ± 0.9; PEG-OB group: 7.0 ± 0.8	PEG-OB, 80 mg/once a weekly subcutaneous injection in combination with a very-low-energy diet (VLED)	• reduction in appetite after 46 day and significant weight loss of 2.8 kg more than the placebo group• body composition, energy expenditure, and metabolic variables differences were non-significant when comparing the PEG-OB group with the placebo group	(133)
a randomized, double-blind, placebo-controlled, multicenter study in obese adults over 3diet+12 treatment weeks	284 overweight and obese; 187 women; 97 men		r-metHuLeptin, 10mg twice daily or 20mg once (a.m. or p.m.) daily as a subcutaneous injection in addition to a mildly energy-restricted diet	• no significant weight loss differences between the obesity and placebo groups• nocturnal administration of r-metHuLeptin have no specific effect on weight loss	(134)
a randomized, placebo-controlled trial in obese subjects with newly diagnosed type 2 diabetes over 2 weeks	18 obese; 6 from 18-placebo; 6 from 18-low dose of leptin (30mg/day) 6 from 18-high dose of leptin (80mg/day)	Placebo group: 27 ± 7; Low dose of leptin group: 24 ± 8; High dose of leptin group: 35 ± 10	r-metHuLeptin, low-dose (30 mg/day), or high-dose (80 mg/day)	• body weight and body composition did not change after 2 weeks of treatment• treatment with• either low-dose or high-dose r-metHuLeptin did not improve• liver, skeletal muscle, or adipose tissue insulin sensitivity• in weight stable, obese subjects with type 2 diabetes.	(135)
a randomized, double-blinded, placebo–a controlled trial, in obese diabetic subjects over 16 weeks	71 obese; 41 men; 30 women	Placebo group: 38.0 ± 6.4; Free leptin in placebo group: 15.8 ± 3.3; Leptin group: 35.2 ± 3.5; Free leptin in leptin group: 22.6 ± 4.7	metreleptin, 10 mg twice daily as a subcutaneous injection	• body weight and circulating inflammatory • markers did not change• HbA1c was marginally reduced• total leptin, leptin-binding protein, and antileptin• antibody levels increased, limiting free leptin availability• and resulting in circulating free leptin levels of ~50 ng/mL	(136)
a randomized, double-blind, placebo-controlled cross-over study, in at least 18 months post- RYGB women who lost on average 30.8% of their pre-surgical body weight over 16 weeks	27 women 13 from 27-placebo; 14 from 27-leptin	Placebo group: 26.1 ± 2.8; Leptin group: 25.1 ± 2.8	metreleptin, 0.05 mg/kg body weight twice daily as a subcutaneous injection	• no significant effect of leptin treatment on body weight in women with relative hypoleptinemia after RYGB	(137)
clinical proof-of-concept study, randomized, double-blind, active-drug-controlled, multicenter study enrolled in overweight/obese subjects over 24-week	177 obese or overweight men and women	treatment with metreleptin (5mg b.i.d.) + placebo for pramlintide (designated as the metreleptin arm), pramlintide (360 μg b.i.d.) + placebo for metreleptin (designated as the pramlintide arm), or pramlintide (360 μg b.i.d.) + metreleptin (5mg b.i.d.) (designated as the pramlintide/metreleptin arm)	pramlintide (analog of human amylin) + r-metHuLeptin	• combined therapy amylin + leptin agonism results in more weight loss in subjects with obesity than either treatment alone and may have therapeutic utility as part of an integrated neurohormonal approach to obesity pharmacotherapy	(138, 139)
a randomized, placebo-controlled trial in overweight and obese subjects with low (baseline) BL leptin (females, ≤16 ng/ml; males, ≤5 ng/ml) over 24 weeks	267 overweight or obese men and women; 171 female; 96 male; Placebo- 111; Metreleptin 10 mg-74; Metreleptin 20 mg-72	171 female mean [SD] BL leptin, 14.2 [13.3]	Metreleptin 10 mg or metreleptin 20 mg as a subcutaneous injection	• Both metreleptin doses decreased weight over time among subjects with low BL leptin;• metreleptin 20 mg showed statistically significant decreases of weight by week 8 (p<0.1)	(140)

r-metHuLeptin, recombinant methionyl human leptin; PEG-OB, pegylated human recombinant leptin; RYGB, Roux-en-Y gastric bypass.

Similarly, Bartness et al. found that Fc-leptin’s weekly administration (engineered leptin) did not lead to weight loss than a placebo group (141). Hukshorn et al. investigated leptin’s influence in combination with a very low-calorie diet using PEG-OB treatment (80 mg administered weekly). They found that PEG-OB treatment resulted in significant additional weight loss in severely energy-restricted, overweight men. It suggests that a decrease in leptin concentrations during starvation increases appetite in humans (133). Also, Zelissen et al. carried out a study with calorific intake restricted to 500 kcal/day coupled with 10 mg of recombinant leptin administered daily (once or twice) for 12 weeks (134). This trial did not show significant weight loss differences between groups with obesity and placebo groups (134). Mittendorfer et al. conducted a clinical study to determine whether leptin treatment has weight loss–independent effects on insulin action in obese subjects with type 2 diabetes. They evaluated the impact of a low and high dose of r-metHuLeptin treatment on insulin action, glucose uptake, and lipolysis (135). The study results showed that r-metHuLeptin does not have weight-loss–independent, clinically important effects on insulin sensitivity in obese subjects with newly diagnosed type 2 diabetes (135).

Furthermore, r-metHuLeptin/metreleptin treatment did not alter body weight or circulating inflammatory markers but marginally reduced HbA1c in obese hyperleptinemic patients with type 2 diabetes (136). Also, total leptin, leptin-binding protein, and antileptin antibody levels increased, limiting free leptin availability (136). Korner et al. investigated whether leptin treatment to post-Roux-en-Y gastric bypass (RYGB) patients promotes further weight loss and shows no significant effect of leptin treatment on women’s body weight with relative hypoleptinemia after RYGB (137). Also, no changes were shown in percent fat mass, resting energy expenditure, thyroid hormones, or cortisol levels (137). A few clinical trials have reported a reduced tendency to regain weight after caloric restriction or weight loss coupled with recombinant leptin’s daily administration. Those studies examined effects on skeletal muscle and autonomic and neuroendocrine adaptation to mass body maintenance (142) and reproductive hormonal regulation (143). Potential mediators of weight regain, including the cortisol, growth hormone, and thyroid axes were not systematically affected (144–147).

Synergistic effects of leptin and amylin promote weight loss while preventing the compensatory reduction in energy expenditure associated with weight loss (138, 148). The combined therapy of leptin and pramlintide (an amylin analog) results in more weight loss in subjects with obesity than either treatment alone. This effect seems to be additive rather than synergistic, suggesting that amylin and its analog cannot increase leptin sensitivity (138, 139). The signaling pathways induced by leptin and amylin overlap and exert an additive effect in humans’ peripheral tissues (149).

Ravussin et al. administered metreleptin and pramlintide to 177 subjects with obesity, which resulted in a mean weight loss of 12.7% after 20 weeks (139). Unfortunately, some subjects developing anti-metreleptin antibodies that led to suspending the study. Later Chan et al. carried out a larger clinical trial with metreleptin and pramlintide on 579 patients with obesity and 134 patients with lipodystrophy for 20-52 weeks (150). Antibody development in patients with obesity or lipodystrophy was associated with higher leptin concentration, and higher antibody titers were associated with higher leptin concentration (150). Other studies have shown that exercise increases leptin sensitivity in human skeletal muscle (151), which may provide an alternative to pharmacological sensitizers.

Despite the remarkable results of leptin-based therapy on weight loss in genetically predisposed obese subjects (mutations in the leptin gene), this approach has a limited or completely absent effect on weight loss in subjects with common obesity, especially in hyperleptinemic patients (3, 152). Different responses to leptin-based therapy on weight loss in obese subjects in clinical studies may be explained by differences in treated population, study design, and administered therapy (leptin type, dosage, etc.). Also, leptin resistance and increased blood leptin level are significant factors that influence leptin-based therapy’s success (3, 152). Indeed, further clinical trials are needed to assess the selectivity and effectiveness of leptin-based therapy on weight loss regarding obesity, particularly defined the threshold of endogenous leptin level as a predictive factor for therapy response to determining the dose-response ratio of leptin-based therapy.

### Development of New Leptin-Based Therapies for Obesity

As previously mentioned, leptin administration combined with a leptin sensitizer is a potential pharmacological strategy for weight loss (5, 6). To avoid difficulties associated with leptin’s short half-life and low stability, leptin analogs capable of binding and activating LEP-R are often used as another approach (6). A few studies have examined blocking negative regulators of the leptin signaling pathway, including SOCS3 and PTP1B, to enhance leptin administration effects in individuals with obesity (5, 153, 154). Inhibitors of PTP1B, such as thiazolidinedione and trodusquemine, suppress weight gain and decrease food intake and body weight in DIO mice (155).

Thus, modulation of endocytosis and the intracellular trafficking of LEP-R (6) may be ways to treat obesity. Leptin must cross the BBB through a specific and saturable transporter (156) to bind LEP-R in the hypothalamus. In obesity, high leptin levels lead to leptin resistance, which the transporter’s hyperactivation may cause by the high levels of leptin (6). Thus, another possible way to improve leptin therapy is to enhance its ability to cross the BBB, potentially fusing it with another molecule to improve uptake by vesicular endocytosis (6).

Although leptin reduces food intake and body mass and stimulates energy expenditure, obese subjects that develop leptin resistance did not respond to leptin-based clinical therapies (157, 158). However, several leptin-sensitizing compounds have been described to influence leptin action and promote beneficial effects in DIO hyperleptinemic mice (159–163). Leptin-sensitizing compounds may be divided into two groups (160). Compounds that enhance the anorectic effect of exogenous leptin but minimally affect weight loss, including meta-chlorophenylpiperazine (164), metformin (165), and betulinic acid (166). The other group comprises compounds that induce weight loss in obese animals with hyperleptinaemia and restore endogenous leptin signaling, such as glucagon-like peptide-1 (167) and heat shock protein 90 inhibitors (168, 169). Some of these leptin sensitizers are in clinical use for diabetes therapy, such as amylin and pramlintide, that enhance leptin action, probably increasing IL-6 production in microglia ventromedial hypothalamic nucleus that in turn activates pSTAT3 signaling in LepR neurons (170, 171). It was found that resveratrol attenuates the expression of leptin in adipocytes, elevates phosphorylation of STAT3 in the hypothalamus, and restores leptin resistance in adult offspring from HF rat mothers attenuating obesity (172). Ozcan and colleagues identified the natural compound celastrol as a potential leptin sensitizer and anti-obesity agent (161). They found that celastrol suppresses food intake, increases energy expenditure, and reduces body weight up to 45% in hyperleptinemic DIO mice (161). Although celastrol’s molecular mechanism regulates leptin sensitivity remains obscure, it was found that celastrol mediates leptin sensitization and exerts anti-obesity effects through increasing interleukin-1 receptor 1 (IL1R1) expression in the hypothalamus (173). Furthermore, celastrol promotes leptin sensitivity through inhibition of 6-phosphofructokinase (PFK) in skeletal muscle and activation of adenosine 5’monophosphate-activated protein kinase (AMPK), which leads to alterations in energy demand from glycolysis to the free fatty acid oxidation in skeletal muscle and increases energy expenditure (174). Another natural compound that acts as a potential leptin sensitizer with additional anti-diabetic actions is withaferin A (162). Treatment of DIO mice with withaferin A reduces body mass by 23%, fat mass by 35%, endoplasmic reticulum stress, hepatic steatosis, leptin level in the blood, and increases the potency of leptin and energy expenditure (162). These effects of withaferin A are exerted at least partly by sensitizing LEP-R signaling and increasing STAT3 phosphorylation in the hypothalamus of DIO mice (162). A partial reduction of plasma leptin level by leptin neutralizing antibody in obesity state improved leptin sensitivity and effectively led to weight loss and enhanced insulin sensitivity (159). Despite the impressive leptin sensitizing effects, using celastrol or withaferin A as an anti-obesity drug has some adverse effects (175–177). Also, these compounds minimally affect the body weight and metabolic disorders in genetically predisposed obesity, such as *ob/ob* and *db/db* mice, which lack leptin or the LEP-R (162).

Zhao et al., using leptin neutralizing antibodies in diverse mouse models, reported that hyperleptinemia triggers developing metabolic diseases (178). Partial leptin reduction has been characterized by returning leptin sensitivity in the hypothalamus, improving insulin sensitivity, and successfully diminishing weight gain (178). The same author suggested that increased leptin sensitivity resulting from partial leptin reduction is a new promising therapeutic tool for treating obesity (178). Another study by Ottaway et al. treated lean and obese mice with an antagonist of the leptin receptor. Regarding (diet-induced obese) DIO mice, antagonist improved body weight (BW) and feeding in lean mice (179). This improvement is related to the decline of expression of Socs3 in the hypothalamus (179). There is an estimation that DIO mice that have hyperleptinemia maintain leptin-feeding inhibition similar to lean mice and oppose an attitude that the stability of DIO in mice is based on resistance to endogenous leptin action (179).

## Conclusions

The discovery of leptin has provided new insight into how to control obesity. The altered expression of leptin and its receptor leads to leptin resistance, which plays a critical role in obesity-related complications (3, 4). Despite knowing that leptin is one of the principal suppressors of appetite and leptin’s link with obesity, the treatment of obesity using leptin-based therapeutics remains to be fully explored (3, 4). The focus of further studies should be identifying new mechanisms of leptin regulation at the whole-body level to design new drugs that reverse leptin resistance. In this regard, understanding the pathogenesis of obesity-related disorders and the regulation of energy homeostasis by leptin should provide new alternatives in obesity treatment.

## Author Contributions

MO, ES-M, and ERI designed, wrote and supervised the manuscript. SS, ME, and SA wrote the manuscript. AJS and TG critically revised the manuscript. All authors contributed to the article and approved the submitted version.

## Funding

This work is part of the collaboration between the Department of Radiobiology and Molecular Genetics, “VINČA” Institute of Nuclear Sciences - National Institute of the Republic of Serbia, University of Belgrade, Belgrade, Serbia, and Computational Bioscience Research Center (CBRC) at King Abdullah University of Science and Technology (KAUST). This work was funded by the Ministry of Education, Science and Technological Development of the Republic of Serbia (Contract No#451-03-9/2021-14/200017) and KAUST grant OSR#4129 (awarded to EI and V.B.B.), which also supported MO and ES-M. ME has been supported by the KAUST Office of Sponsored Research (OSR) Award no. FCC/1/1976-17-01, and TG by the King Abdullah University of Science and Technology (KAUST) Base Research Fund (BAS/1/1059-01-01).

## Conflict of Interest

The authors declare that the research was conducted in the absence of any commercial or financial relationships that could be construed as a potential conflict of interest.

## Abbreviations

AgRP, agouti-related protein; AMPK, adenosine 5’monophosphate-activated protein kinase; ARC, arcuate nucleus; BAT, brown adipose tissue; BBB, blood-brain barrier; CCL2, CC-chemokine ligand 2; CD, cluster of differentiation; CNS, central nervous system; CSF-3, colony-stimulating factor 3; DIO, diet-induced obese; DMH, dorsomedial hypothalamic nucleus; FN III, fibronectin type III; GH, growth hormone; IL, interleukin; IL1R1, interleukin 1 receptor 1; JAK, Janus-associated kinase; LEP-R, leptin receptor; LEP-Rb, LEP-R isoform b; LH, lateral hypothalamic nuclei; LIF, leukemia inhibitory factor; MAPK/ERK - mitogen-activated protein kinases/extracellular signal-regulated kinase; MCR, melanocortin receptor; NPY, neuropeptide Y; NTS, nucleus tractus solitarius; ob, obese gene; PFK, 6-phosphofructokinase; PI3K, phosphoinositol-3 kinase; POMC, proopiomelanocortin; POA, preoptic area; pQTL, protein quantitative trait locus; PTP1B, protein tyrosine phosphatase 1B; PVNparaventricular nucleus; RYGB, Roux-en-Y gastric bypass; SLR, selective leptin resistance; SOCS3, cytokine signaling 3; STAT, signal transducer and activator of transcription; TNFα, tumor necrosis factor α; VMH, ventromedial hypothalamic nucleus; VTA, ventral tegmental area; WHO, World Health Organization; α-MSH, alpha-melanocyte-stimulating hormone.
